# Polarity correspondence effect between loudness and lateralized response set

**DOI:** 10.3389/fpsyg.2015.00683

**Published:** 2015-05-22

**Authors:** Seah Chang, Yang Seok Cho

**Affiliations:** Laboratory of Human Performance, Department of Psychology, Korea UniversitySeoul, South Korea

**Keywords:** polarity coding, auditory stimulus–response compatibility, response selection, SMARC effect, SPARC effect

## Abstract

Performance is better when a high pitch tone is associated with an up or right response and a low pitch tone with a down or left response compared to the opposite pairs, which is called *the spatial-musical association of response codes* effect. The current study examined whether polarity codes are formed in terms of the variation in loudness. In Experiments 1 and 2, in which participants performed a loudness-judgment task and a timbre-judgment task respectively, the correspondence effect was obtained between loudness and response side regardless of whether loudness was relevant to the task or not. In Experiments 3 and 4, in which the identical loudness- and timbre-judgment tasks were conducted while the auditory stimulus was presented only to the left or right ear, the correspondence effect was modulated by the ear to which the stimulus was presented, even though the effect was marginally significant in Experiment 4. The results suggest that loudness produced polarity codes that influenced response selection (Experiments 1 and 2), and additional spatial codes provided by stimulus position modulated the effect, generating the stimulus eccentricity effect (Experiments 3 and 4), which is consistent with the polarity correspondence principle.

## Introduction

Performance is better when stimulus and response alternatives spatially correspond with each other than when they do not. Left–right responses are faster and more accurate when the locations of stimulus and response are compatible than when they are not, regardless of whether stimulus location is relevant to the task or not (Eimer et al., 1995; Lu and Proctor, 1995). As an example, the spatial stimulus–response compatibility (SRC) effect obtained when the target location is irrelevant to the task is called the *Simon effect* (Simon and Rudell, 1967). Furthermore, the SRC effects are also found with orthogonal arrays of stimulus and response sets. When a vertically arrayed stimulus set is coupled with a horizontally arrayed response set, an up-right/down-left advantage is obtained (Weeks and Proctor, 1990; Lippa and Adam, 2001). These various kinds of spatial SRC effect have been thought to be due to spatial coding of the stimulus and response alternatives (Umiltá and Nicoletti, 1990; Hommel, 1997). The spatial coding accounts suggest that spatial codes are formed with respect to multiple frames of reference, resulting in correspondence effects between stimulus and response spatial codes (Lamberts et al., 1992; Hommel and Lippa, 1995; Roswarski and Proctor, 1996; Adam et al., 1998; Cho and Proctor, 2003).

The SRC effect has been obtained even with a set of stimuli with no obvious spatial feature. For instance, when participants make an odd–even parity judgment of a digit stimulus from the set of 0–9, performance is better when the left response is made to a small number and the right response to a large number, which is the phenomenon called *the spatial-numerical association of response codes (SNARC)* effect. The SNARC effect has been attributed to a mental number line with space in which small numbers are encoded to the left side and large numbers to the right side (Dehaene et al., 1993). The mapping of up or right response to a high pitch tone and down or left response to a low pitch tone typically yields better performance than the opposite mapping. This type of SRC effect is referred to as *the spatial musical association of response codes (SMARC)* effect (Rusconi et al., 2006), or alternatively, as *the spatial pitch association of response codes (SPARC)* effect (Lidji et al., 2007). The SMARC effect is manifested by faster reaction times (RTs) with the mapping of high pitch tone to up or right response and low pitch tone to down or left response than the opposite mapping. The SMARC effect has been found even when the pitch height of tones was task-irrelevant while the timbre of the tone (Rusconi et al., 2006; Lidji et al., 2007; Cho et al., 2012) or the color of a visual stimulus (Nishimura and Yokosawa, 2009; Cho et al., 2012) was task-relevant.

The vertical SMARC effect has been consistently obtained across studies, irrespective of task relevance of pitch height and musical proficiency. For example, when Rusconi et al. (2006) conducted a pitch comparison task in which the pitch height of a target tone was judged in comparison to a referent tone, both musician and non-musician participants showed better performance when an up response was made to a high pitch tone and a down response to a low pitch tone than the opposite mapping. Moreover, in Rusconi et al.’s (2006) Experiments 2 and 3, and Lidji et al.’s (2007) Experiment 3, musician and non-musician participants performed a timbre judgment task in which the timbre of a target tone was judged. Even when pitch height was irrelevant to the task, performance was better when a high pitch tone was responded to with the up response and a low pitch tone with the down response than the opposite pairs for both non-musician and musician groups in their studies. The results indicated that non-musicians as well as musicians associated high pitch tones with the up response and low pitch tones with the down response, resulting in the vertical SMARC effect, regardless of the task relevance.

The SMARC effect was also found when a lateralized response set was used. Lidji et al. (2007) found that the pairs of “high-right/low-left” produced better performance than those of “high-left/low-right” when non-musician participants were asked to judge whether the pitch of the target tone is higher or lower than the preceding referent tone using left or right response keys in their Experiment 2. Rusconi et al. (2006) also reported a marginally significant 16.5-ms high-right/low-left advantage for non-musicians, in their Experiment 1. However, while consistent results have been obtained on this horizontal SMARC effect when the pitch height of tones was task-relevant, contradictory results have been reported across experiments in which the pitch height was irrelevant to the task. When Rusconi et al. had participants perform a timbre-judgment task in their Experiments 2 and 3, musically trained participants showed a significant horizontal SMARC effect, but musically untrained participants did not. Lidji et al. (2007) also obtained a similar result in their Experiment 1, showing an evident horizontal SMARC effect only for musicians.

To explain the SMARC effect, Lidji et al. (2007) proposed the *spatial mental representation account*. According to this account, high pitch tones are associated with the upper part of the mentally represented vertical pitch line and low pitch tones with the lower part of the line. The association of pitch height with the vertical line seems plausible in the sense that pitch height is verbally expressed with vertical spatial terms such as “high” and “low” in multiple languages, including English, Korean, Chinese, and Spanish. Rusconi et al. (2006) argued that the presence of the vertical SMARC effect is consistent with the view that the pitch height is represented as a multidimensional spatial form, as Mudd (1963) demonstrated, suggesting that “human cognitive system maps pitch onto a mental representation of space” (Rusconi et al., 2006, p. 126). Moreover, Lidji et al. (2007) suggested that the correspondence effect between pitch height and vertical response is due to an automatic activation of the association between them, irrespective of musical experience. When people respond with vertically aligned response sets, high pitch tones are automatically associated with the up response and low pitch tones with the down response. Furthermore, Lidji et al. (2007) attributed the significant horizontal SMARC effect in musicians to the familiarity with piano keyboard which locates lower tones on the left side and higher tones on the right side. The participants’ knowledge of the keyboard structure or the usual order of singing or playing the musical notes, which is activated only when the pitch is explicitly processed in non-musicians, is a crucial factor for the emergence of the horizontal SMARC effect. Lidji et al. (2007) suggested that the lack of the SMARC effect was due to non-musicians being not able to automatically associate pitch to the horizontal spatial representation when pitch was irrelevant to the task. Because Lidji et al. (2007) attributed the absence of the SMARC effect to the inability to represent pitch to spatial representation, the comparison between musicians and non-musicians was necessary.

On the contrary, Nishimura and Yokosawa (2009) found a significant 8-ms horizontal SMARC effect when participants performed a visual task accompanied by accessory high or low pitch tone to the left or right ear. They suggested that the musical experience of a short period of time might have been sufficient to form the spatial-musical association because the participants experienced musical education only in elementary and junior high schools as a regular course. Recently, Cho et al. (2012) suggested that a referent tone is crucial for non-musicians to categorically code pitch height automatically by indicating that a referent tone was presented only when pitch height was relevant to the task in both Rusconi et al.’s (2006) and Lidji et al.’s (2007) experiments. To examine this possibility, Cho et al. (2012) had participants perform a pitch-judgment task, a timbre-judgment task, and a color discrimination task with or without a referent tone. The results dissociated the influence of a referent tone on the horizontal SMARC effect from the influence of the task relevance variable. When pitch height was task-relevant in Experiment 1, non-musicians showed a significant horizontal SMARC effect regardless of the presence of the referent tone, which reveals that they were able to categorically code pitch height even without a referent tone. In contrast, when pitch height was irrelevant to the task, non-musician participants produced a horizontal SMARC effect only when a referent tone was provided (Experiments 2 and 3), while musicians produced it regardless of the presence of a referent tone (Experiment 2).

In line with Cho et al.’s (2012) recent findings, Rusconi et al. (2006) and Nishimura and Yokosawa (2009) suggested that the horizontal SMARC effect might be a variant of the orthogonal SRC effect and that the SMARC effect is due to *polarity correspondence*. According to the polarity correspondence principle, initially proposed by Proctor and Cho (2006), stimuli and responses are coded as either + or - polarity relative to a reference point across multiple dimensions. The summed polarity codes for the stimulus and response contribute to the response selection efficiency in various binary choice tasks. That is, binary choice performance is better when the polarity code of the stimulus corresponds to the polarity code of the response in comparison to when they do not. As demonstrated in the orthogonal SRC effect, “up” and “right” are coded as + polarity and “down” and “left” are coded as - polarity, resulting in the up-right/down-left advantage. Similarly, the SMARC effect arises because high pitch and up/right response are coded as + polarity whereas low pitch and down/left response as - polarity. Cho et al. (2012, p. 733) further argued that “the horizontal SMARC effect occurs when the verticality is task-relevant or when a referent tone is presented relative to which pitch height is coded categorically”.

In general, recent studies on auditory SRC effects tend to address the question of pitch representation. Even though a tone is a multidimensional stimulus that consists of pitch, loudness, and timbre dimensions, little is known about how loudness is mentally represented and how these mental representations are used in the response selection process. It is important to note that loudness also has a categorical characteristic which can be divided into low-level loudness (soft) and high-level loudness (loud) relative to a reference, enabling categorical representation, and relative judgments. Therefore, if loudness is also coded categorically relative to a reference, a correspondence effect with binary responses would be obtained (Proctor and Cho, 2006). Furthermore, it has been found that pitch and loudness dimensions are inseparable and integral, in that making a variation on one dimension causes interference with the categorizing process on the other dimension (Grau and Kemler-Nelson, 1988; Marks, 1989; Melara and Marks, 1990a,b,c; Melara et al., 1992). Therefore, investigating the underlying mechanism of loudness coding and its influence on response selection process would provide useful insights for previous study results on auditory SRC effects using pitch.

The primary aim of this research was to examine whether loudness produces a correspondence effect in a lateralized response setting. As predicted by the polarity correspondence principle, if loudness is also categorically coded as + or - polarity relative to the referent tone, performance would be better when the polarity code of loudness corresponds to that of a response alternative than when it did not. To test this, a loudness-judgment task and a timbre-judgment task were conducted respectively in Experiments 1 and 2, with non-musician participants. The secondary aim was to examine whether the correspondence effect is modulated by the stimulus eccentricity. Previously, it has been found that the up-right/down-left advantage increases in size when the response set is positioned at the right hemispace and reverses to an up-left/down-right advantage when it is positioned at the left hemispace. This variation in the orthogonal SRC effects as a function of the response-set location along the horizontal dimension is called the *response eccentricity effect* (Michaels, 1989; Michaels and Schilder, 1991; Weeks et al., 1995; Cho and Proctor, 2002, 2004, 2005; Proctor and Cho, 2003; Nishimura and Yokosawa, 2006). This phenomenon occurs because response location provides an additional spatial code which contributes to the overall polarity of each response alternative (Proctor and Cho, 2006). Similarly, it was assumed that if the additional spatial code is provided by stimulation position, the correspondence effect would be modulated by stimulation position, which can be referred to as the stimulus eccentricity effect. To examine this hypothesis based on the polarity correspondence principle, Experiments 3 and 4 investigated the eccentricity effect by conducting a loudness-judgment task and a timbre-judgment task while the stimulation position to which the sound was provided was manipulated. All tone stimuli were manipulated in terms of loudness level while their frequencies were kept at a constant 1,000 Hz which has been a commonly used frequency for reference to which other frequency tones are adjusted in previous studies (e.g., Howard and Angus, 2009).

## Experiment 1: Loudness-Judgment Task

The purpose of Experiment 1 was to examine whether the loudness of sound produces a correspondence effect with a lateralized response set when participants perform a loudness-judgment task in which the loudness of sound was task-relevant. Participants were to discriminate whether the loudness of a pure tone was louder or softer than a referent tone. Since the loudness of tones was relevant to the task, the correspondence effect between loudness and response side was expected if the loudness was coded in terms of its polarity. If polarity codes are formed in terms of loudness, a high-level loudness tone following a reference would be coded as + polarity and a low-level loudness tone following the referent would be coded as - polarity. Therefore, the polarity correspondence with right response (+) and left response (-) would generate a loud-right/soft-left advantage.

### Materials and Methods

#### Participants

As did the previous SMARC research (Lidji et al., 2007), 16 undergraduate students (mean age = 22.13, 10 females) at Korea University participated for monetary reward of KRW 5,000 (about 4 US dollars). Four had no prior musical training experience; the other 12 had an average of 4.58 years of musical training and had stopped it since 12.91 years of age on average. All were right-handed and had normal hearing as determined by self-report. The present and following experiments were approved by the Institutional Review Board at Korea University (KU-IRB-13-25-A-1).

#### Apparatus, Stimuli, and Procedure

All experiments were programmed and presented using E-prime software (Version 2.0, Psychology Software Tools, Inc.). Stimuli were presented on a 17-inch CRT monitor of a personal computer and viewed at a distance of approximately 60 cm. Responses were made by pressing the leftmost or rightmost key among five keys on a Micro Experimental Laboratory 2.0 response box with the left and right index fingers.

**FIGURE 1:**
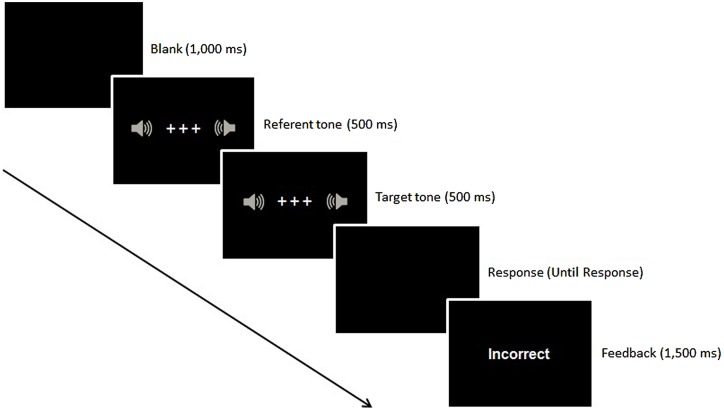
**Example of a trial sequence in Experiments 1 and 2**.

Three white crosses (0.4° × 0.4°, see **Figure 1**) were presented against a black background as fixation. The imperative stimuli were a low-level loudness pure tone (59-dB, SPL) and a high-level loudness pure tone (77-dB, SPL) which were given to the participants through PC convertible headphones. An intermediate-level loudness pure tone (67-dB, SPL) was used as the reference. Loudness for each tone was measured at the same location on the same day in a soundproof booth. All different loudness tones were generated for the same pitch (1,000 Hz).

Participants were tested individually in a soundproof booth with dim light. They were instructed to align their body midline with the center of the screen and put each index finger on the left-most and right-most keys of the response box, which was lined up with the center of the screen. They were asked to press the left or right key to the low- or high-level loudness of each target auditory stimulus as quickly as possible while maintaining high accuracy. The experiment consisted of two sessions of 16 practice trials and 160 test trials each. Participants performed the loudness-judgment task with one mapping of low- and high-level loudness to left and right responses in the first session and the other mapping in the second session, with the order counterbalanced across participants.

At the beginning of each trial, the fixation crosses were presented at the center of the screen for 500 ms. The referent tone was presented simultaneously with the fixation crosses for all trials. The imperative auditory stimulus was presented for 500 ms, followed by a dark screen that remained until a response was made. The word “Incorrect” in white during the practice trials and in red during the test trials was displayed for 500 ms as feedback at the center of the screen when an incorrect response was made. The white fixation for the next trial appeared 500 ms after the correct response or the error feedback. One minute rest period was given between the sessions.

### Results

Reaction times shorter than 125 ms and longer than 1,250 ms were excluded as outliers (0.16%). Mean correct RT and percentage of error (PE) for each participant were calculated as a function of loudness (low- or high-level loudness) and response side (left or right). Repeated measures analyses of variance (ANOVAs) were conducted on the mean RT and PE data, with those variables as within-subject factors. Mean RT and PE data are shown in **Table 1**.

**Table 1:** Mean reaction time (RT, in milliseconds), percentage of error (PE), and stimulus and response polarities in parenthesis in Experiment 1 as a function of loudness and response side.

Response side	Low-level loudness		High-level loudness	
	RT (SD)	PE (SD)	RT (SD)	PE (SD)
Left	357 (49.42)	0.47 (0.90)	357 (63.61)	2.50 (3.16)
	(-,-)		(+,-)	
Right	364 (51.46)	2.20 (2.61)	329 (59.36)	2.66 (2.41)
	(-,+)		(+,+)	
Correspondence effect	7	1.73	28	-0.16

#### RT Analysis

**FIGURE 2:**
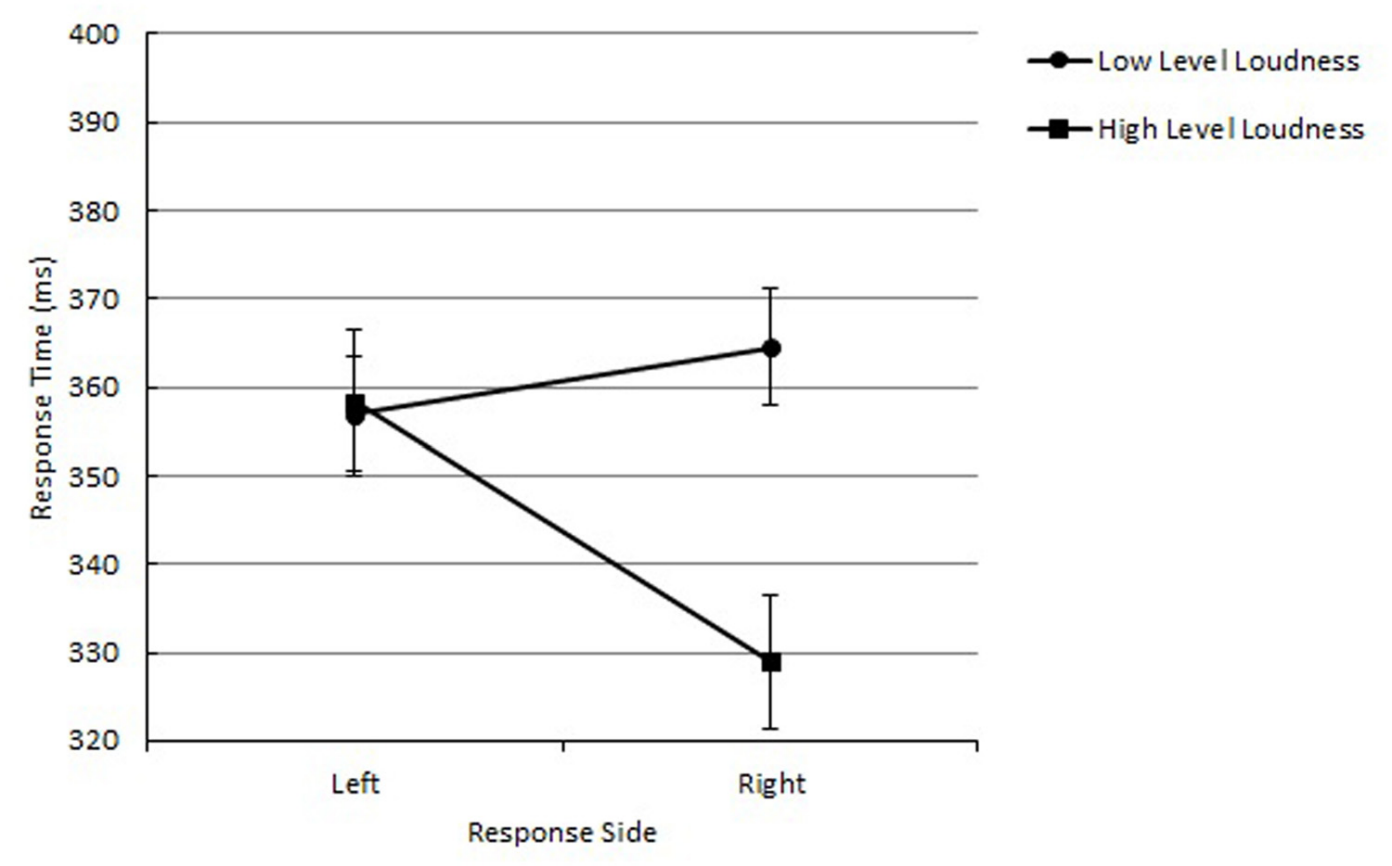
**Mean reaction times (RTs) as a function of loudness and response side in Experiment 1 are shown along with their SE**.

The main effect of loudness was significant, *F*(1,15) = 7.95, *p* = 0.0130, *MSE* = 628, $\eta_{p}^{2}$ = 0.23. The mean RT was shorter for high-level loudness tones (*M* = 343 ms) than low-level loudness tones (*M* = 361 ms). The main effect of response side was also significant, *F*(1,15) = 6.15, *p* = 0.0255, *MSE* = 293, $\eta_{p}^{2}$ = 0.10, showing that responding with the right hand (*M* = 346 ms) was faster than with the left hand (*M* = 357 ms). Of importance, a significant interaction between loudness and response side was obtained, *F*(1,15) = 4.76, *p* = 0.0454, *MSE* = 1,098, $\eta_{p}^{2}$ = 0.24, indicating a correspondence effect between loudness and response side. The mean RT was shorter with loud-right/soft-left mapping (*M* = 343 ms) than loud-left/soft-right mapping (*M* = 361 ms; see **Figure 2**).

#### PE Analysis

The overall PE was 1.96%. The main effect of loudness was significant, *F*(1,15) = 6.75, *p* = 0.0202, *MSE* = 4, $\eta_{p}^{2}$ = 0.44, indicating that PE was higher for the high-level loudness (2.58%) than the low-level loudness (1.33%). The main effect of response side did not reach significance. Of importance, the interaction between loudness and response side reached significance, *F*(1,15) = 4.64, *p* = 0.0480, *MSE* = 2, $\eta_{p}^{2}$ = 0.24. The PE was lower with loud-right/soft-left mapping (1.56%) than loud-left/soft-right mapping (2.35%).

### Discussion

As in pitch-judgment experiments (Rusconi et al., 2006; Lidji et al., 2007; Cho et al., 2012), the correspondence effect between loudness and response side was obtained in the loudness-judgment task: the performance of the loudness-judgment task was better when participants performed the task with the loud-right/soft-left mapping than with the loud-left/soft-right mapping, resulting in an 18-ms loud-right/soft-left advantage.

As shown in **Figure 2**, the loud-right mapping elicited a larger effect than the soft-left mapping. This is probably due to additional processing benefits of the + polarity codes themselves, as Lakens (2012) suggested. The presence of both main effects in the stimulus (loudness) and response dimensions (response side) indicates processing advantages of + polarity in both dimensions, while the polarity correspondence effect occurs when two polarity codes correspond. Overall, the obtained results are in line with the polarity correspondence principle (Proctor and Cho, 2006). Like the pitch of tones (see Cho et al., 2012), relative loudness was also categorically coded based on the referent tone when participants were required to process the loudness explicitly to perform the task, resulting in a polarity correspondence effect with the response polarity codes. One could argue that this correspondence effect was due to the sound intensity effect in which choice response time decreases as the sound intensity increases up to the intensity level of 85 dB (van der Molen and Keuss, 1979, 1981; Keuss and van der Molen, 1982; Jaśkowski et al., 2009). However, the lack of the sound intensity effect for the left response indicates that the obtained mapping effect was due to polarity correspondence between loudness and location of responses.

## Experiment 2: Timbre-Judgment Task

In Experiment 1, the correspondence effect between loudness and response side was found when loudness was relevant to the task. This result is in agreement with the findings of the previous SMARC experiments in which pitch height was relevant to the task. Loudness, as well as, pitch height is categorically coded when participants responded explicitly. The aim of Experiment 2 was to investigate whether the correspondence effect is obtained when loudness is irrelevant to the task. In Experiment 2, participants performed a timbre-judgment task in which they responded to whether a target tone was a piano tone or a violin tone. For non-musicians to be able to create categorical codes for irrelevant loudness automatically, a pure tone with intermediate-level loudness was provided as a reference. In this task, the loudness was perceived by participants implicitly. If the categorical codes for the loudness are formed relative to the referent tone even when the loudness is irrelevant to the task, the correspondence effect would be present.

### Materials and Methods

#### Participants

Sixteen new undergraduate students (mean age = 22.06, 11 females) at Korea University participated for payment of KRW 5,000 (about 4 US dollars). Two had no prior musical training experience; the other 14 had an average of 6.35 years of musical training and had stopped it since 12.86 years of age on average. All were right-handed and had normal hearing as determined by self-report.

#### Apparatus, Stimuli, and Procedure

The apparatus, stimuli, and procedure were identical to Experiment 1, except as noted. The stimuli consisted of a low-level loudness tone (58-dB, SPL) and a high-level loudness tone (77-dB, SPL). Each tone was synthesized with piano and violin timbre for a total of four different stimuli. The target tones were given to the participants through PC convertible headphones. The referent tone was an intermediate-level loudness pure tone (66-dB, SPL). Before the experiment began, participants heard the stimuli and determined that they can distinguish the piano and violin tones. The experiment consisted of 16 practice trials and 320 test trials total. Participants performed the timbre-judgment task and timbre-to-response mapping was counterbalanced across participants. Participants were instructed to press one key when a piano tone was presented and the other key when a violin tone was presented, as quickly as possible while maintaining high accuracy.

### Results

0.20% of trials were removed from analyses using the same RT cutoff criteria as in Experiment 1. Mean correct RT and PE were calculated for each participant as a function of loudness (low- or high-level loudness) and response side. ANOVAs were conducted on the mean RT and PE data, with those variables as within variables and timbre-to-response mapping as a between-subject variable. Mean RT and PE data are shown in **Table 2**.

**Table 2:** Mean RT (in milliseconds), PE, and stimulus and response polarities in parenthesis in Experiment 2 as a function of loudness and response side.

Response side	Low-level loudness		High-level loudness	
	RT (SD)	PE (SD)	RT (SD)	PE (SD)
Left	390 (55.65)	2.04 (2.05)	380 (57.78)	3.44 (2.80)
	(-,-)		(+,-)	
Right	387 (55.14)	3.75 (2.19)	360 (47.81)	2.19 (1.80)
	(-,+)		(+,+)	
Correspondence effect	-3	1.71	20	1.25

#### RT Analysis

**FIGURE 3:**
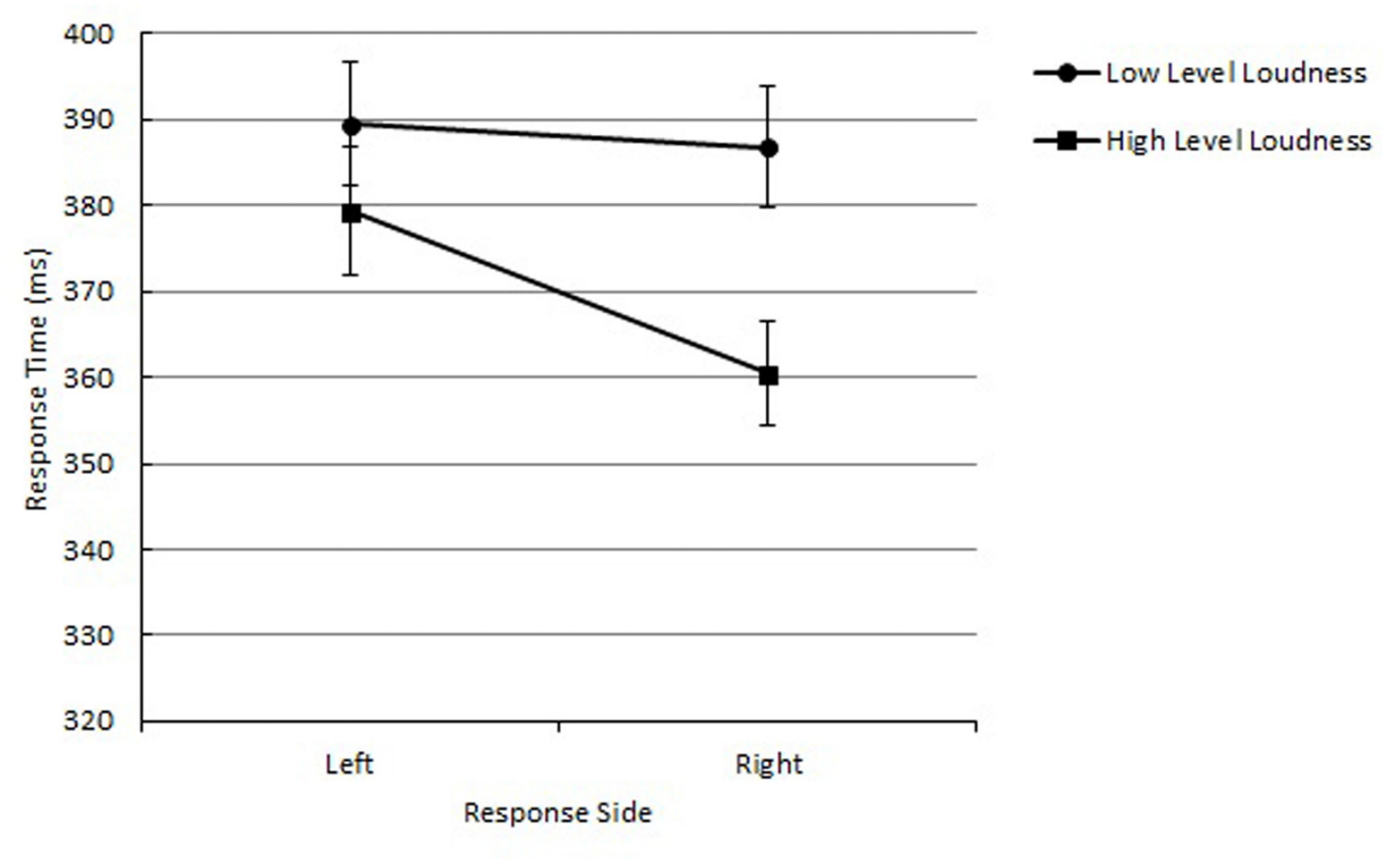
**Mean RTs as a function of loudness and response side in Experiment 2 are shown along with their SE**.

The main effect of loudness was significant, *F*(1,14) = 51.14, *p* < 0.0001, *MSE* = 102, $\eta_{p}^{2}$ = 0.67, reflecting that the mean RT was shorter for high-level loudness tones (*M* = 370 ms) than low-level loudness tones (*M* = 388 ms). The main effect of response side was not significant. Importantly, the interaction between loudness and response side was significant, *F*(1,14) = 5.74, *p* = 0.0311, *MSE* = 184, $\eta_{p}^{2}$ = 0.29. The mean RT was shorter with loud-right/soft-left relation (*M* = 375 ms) than loud-left/soft-right relation (*M* = 383 ms; See **Figure 3**). Any effects regarding timbre-to-response mapping did not reach significance, indicating that it is not a critical factor for the correspondence effect between loudness and response side.

#### PE Analysis

The overall PE was 2.86%. The interaction between loudness and response side was marginally significant, *F*(1,14) = 4.50, *p* = 0.0522, *MSE* = 7.79, $\eta_{p}^{2}$ = 0.24, reflecting that performances were more accurate when high-level loudness was responded to with right hand and low-level loudness with left hand (2.11%) than the opposite (3.60%). The interaction between timbre-mapping and response side was significant, *F*(1,14) = 7.69, *p* = 0.0150, *MSE* = 3, $\eta_{p}^{2}$ = 0.19. When responses were made with left hand, piano-left/violin-right mapping elicited higher error rates (3.34%) than the other mapping (2.11%). But, when responses were made with right hand, piano-right/violin-left mapping elicited higher error rates (3.60%) than the other mapping (2.35%). This effect reflects that piano tones elicited higher error rates than violin tones regardless of responding hands. It is possible that participants confused the piano tones with violin tones because piano tones were created electronically, which made them sound somewhat unnatural. However, even assuming this possibility, this effect is far from the mapping effect, showing that the mapping of piano or violin to responses did not influence the obtained correspondence effect.

### Discussion

The correspondence effect between loudness and response side was evident even when the loudness was task-irrelevant in the RT data: an 8-ms loud-right/soft-left advantage was obtained. As in Experiment 1, an asymmetric effect was observed between loud-right and soft-left mappings, which might have resulted from the additional processing benefits due to the + polarity codes themselves (Lakens, 2012). The finding that the correspondence effect was obtained even when participants were required to focus not on the loudness of sound but on the timbre is in line with the results from Cho et al.’s (2012) Experiment 2 in that loudness was also coded automatically relative to the referent even when the loudness of sound was irrelevant to the task. As in Cho et al.’s (2012) experiment in which pitch height was manipulated, non-musician participants were also able to form the categorical codes for loudness when the referent tone was provided, as implied by the polarity correspondence principle.

The correspondence effect between loudness and response side obtained in Experiments 1 and 2 could have been due to the spatial mental representation account. There is a population stereotype for pulling a control to the right with a horizontal linear control or rotating a knob clockwise to increase loudness (Proctor and Van Zandt, 2008). That is, the existence of the mental loudness line on which loudness is spatially represented from left to right is also plausible. To test this possibility, the effects of an additional polarity code caused by the eccentricity of stimulation position were tested in Experiments 3 and 4. If the obtained correspondence effects were due to polarity coding, the correspondence effect would be modulated when additional spatial codes are provided. In other words, adding extra spatial codes by manipulating stimulation position would influence the overall polarities of spatial codes and modulate the correspondence effect between loudness and response side. On the other hand, the spatial mental representation account does not predict different patterns of results depending on the stimulation position.

## Experiment 3: Loudness-Judgment Task with Eccentricity Manipulated

Experiments 1 and 2 showed that there was a correspondence effect between loudness and response side regardless of whether loudness was task-relevant or not. The purpose of Experiment 3 was to expand the results obtained in the previous experiments and to determine the underlying mechanism of the correspondence effect of loudness with response side by examining whether the correspondence effect is modulated by stimulus position. According to the polarity correspondence principle, the magnitude and direction of the orthogonal SRC effect is expected to vary along the horizontal location at which the responses are made, which is called *the response eccentricity effect* (Michaels, 1989; Weeks et al., 1995; Cho and Proctor, 2002). Because the combined elemental spatial codes determine the overall polarity of each response alternative (Proctor and Cho, 2006), the additional spatial code of response location contributes to an increase or decrease in the magnitude of the polarity correspondence effect. Whereas response position was manipulated in order to add extra spatial codes in all previous experiments, resulting in a response eccentricity effect (Michaels, 1989; Michaels and Schilder, 1991; Cho and Proctor, 2002, 2004, 2005; Proctor and Cho, 2003; Nishimura and Yokosawa, 2006), stimulation position was manipulated in Experiment 3. Because auditory stimuli were given through the headphone in this experiment, manipulating stimulation position to which the sound was given was expected to be a more direct way to generate an additional spatial code than manipulating response position. Just as the eccentricity of response location, if the stimulus eccentricity contributes to addition of an extra spatial code and influences response selection process, the correspondence effect would be modulated by the side to which the target sound was presented. The polarity correspondence principle predicts that the correspondence effect would be larger when the sound is provided to the right ear than when the sound is given to the left ear.

### Materials and Methods

#### Participants

Sixteen new undergraduate students (mean age = 23.06, 11 females) at Korea University participated for monetary reward of KRW 5,000 (about 4 US dollars). Two had no prior musical training experience; the other fourteen had an average of 5.28 years of musical training and had stopped it since 11.57 years of age on average. All were right-handed and had normal hearing as determined by self-report.

#### Apparatus, Stimuli, and Procedure

**FIGURE 4:**
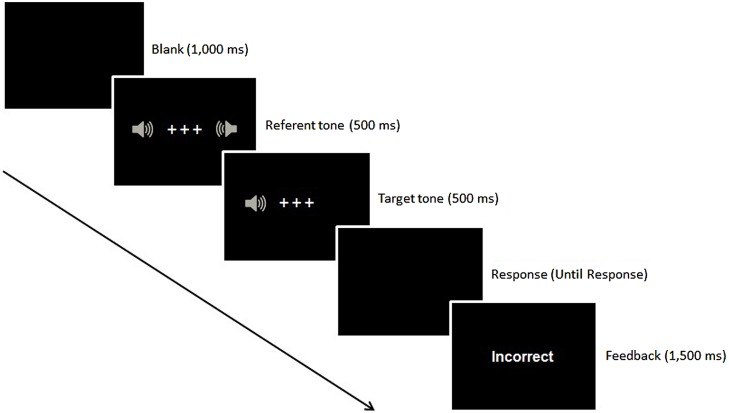
**Example of a trial sequence in Experiments 3 and 4**.

The apparatus, stimuli, and procedure were identical to Experiment 1, except as noted (see **Figure 4**). The stimuli consisted of a low-level loudness pure tone (59-dB, SPL) and a high-level loudness pure tone (77-dB, SPL), which were given to the participants unilaterally through PC convertible headphones. Each tone was presented only to the left or right ear randomly. The referent tone was an intermediate-level loudness pure tone (67-dB, SPL) which was presented bilaterally. The experiment consisted of two sessions of 16 practice trials and 320 test trials each. Participants performed the loudness-judgment task and were told to ignore the location of the sound presented.

### Results

0.12% of trials were removed from analyses using the same RT cutoff criteria as in Experiments 1 and 2. Mean correct RT and PE were calculated for each participant as a function of loudness, response side, and stimulation position. Repeated measures ANOVAs were conducted on the mean RT and PE data, with loudness (low- or high-level loudness), response side (left or right), and stimulation position (left or right) as within-subject factors. Mean RT and PE data are shown in **Table 3**.

**Table 3:** Mean RT (in milliseconds), PE, and stimulus and response polarities in parenthesis in Experiment 3 as a function of loudness, response side, and stimulation position.

	Left ear				Right ear			
Response side	Low-level loudness		High-level loudness		Low-level loudness		High-level loudness	
	RT (SD)	PE (SD)	RT (SD)	PE (SD)	RT (SD)	PE (SD)	RT (SD)	PE (SD)
Left	353 (40.20)	0.55 (1.02)	338 (59.54)	1.88 (3.13)	390 (48.78)	2.90 (3.47)	383 (55.44)	4.62 (2.92)
	(-,+)		(+,+)		(-,-)		(+,-)	
Right	380 (46.61)	3.20 (3.74)	377 (51.32)	3.84 (2.41)	359 (47.50)	1.72 (1.82)	335 (51.73)	1.25 (2.62)
	(-,-)		(+,-)		(-,+)		(+,+)	
Correspondence effect	27	2.66	-39	-1.96	-31	-1.18	48	3.37

#### RT Analysis

**FIGURE 5:**
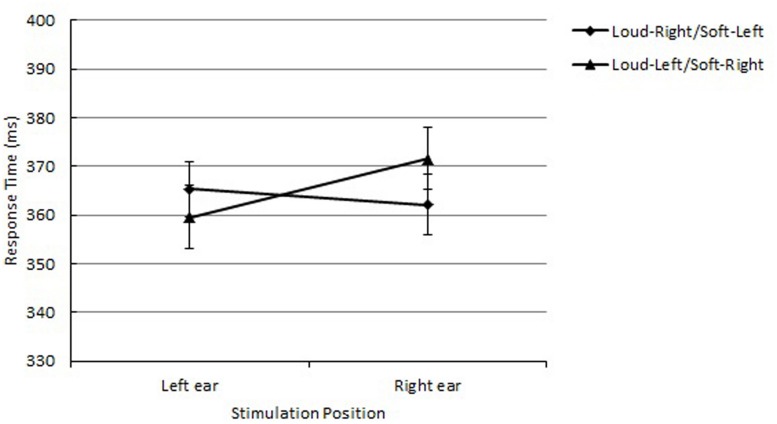
**Mean RTs as a function of loudness-response mapping and stimulation position in Experiment 3 are shown along with their SE**.

The main effect of loudness was significant, *F*(1,15) = 4.59, *p* = 0.0490, *MSE* = 1,067, $\eta_{p}^{2}$ = 0.69. The mean RT was shorter for high-level loudness tones (*M* = 358 ms) than low-level loudness tones (*M* = 371 ms). The main effect of stimulation position was also significant, *F*(1,15) = 4.76, *p* = 0.0454, *MSE* = 130, $\eta_{p}^{2}$ = 0.22. When the sound was heard from the left ear, the mean RT was shorter (*M* = 362 ms) than from the right ear (*M* = 367 ms). Furthermore, the interaction between response side and stimulation position was significant, *F*(1,15) = 117.99, *p* < 0.0001, *MSE* = 352, $\eta_{p}^{2}$ = 0.95, reflecting the auditory Simon effect. The mean RTs were shorter when the sound and response locations corresponded (*M* = 346 ms) when they did not (*M* = 382 ms). The interaction between loudness and response side did not reach significance, *F*(1,15) < 1.0. However, of importance, the interactions of loudness, response side, and stimulation position were significant, *F*(1,15) = 12.36, *p* = 0.0031, *MSE* = 144, $\eta_{p}^{2}$ = 0.45, indicating that the correspondence effect between loudness and response side was found as a function of stimulation position (see **Figure 5**). When the sound was given to the right ear, a 9-ms loud-right/soft-left advantage was obtained, *F*(1,15) = 1.27, *p* = 0.2766, *MSE* = 936. On the other hand, when the sound was provided to the left ear, a 6-ms loud-left/soft-right advantage was obtained, *F*(1,15) < 1.0.

#### PE Analysis

The overall PE was 2.49%. The main effect of loudness was significant, *F*(1,15) = 8.04, *p* = 0.0125, *MSE* = 3, $\eta_{p}^{2}$ = 0.18. PE was higher for the low-level loudness (2.90%) than the high-level loudness (2.09%). The interaction between stimulation position and response side reached significance, *F*(1,15) = 17.46, *p* = 0.0008, *MSE* = 10, $\eta_{p}^{2}$ = 0.64, indicating the auditory Simon effect. When the sound location and response side matched (1.35%), the performance was better than when they did not (3.64%). All the other effects were not significant.

### Discussion

Different patterns of the correspondence effect between loudness and response side were found depending on stimulation position. The loud-right/soft-left advantage was evident when the sound was presented to the right ear (9 ms) but reversed to a loud-left/soft-right advantage when the sound was presented to the left ear (6 ms), resulting in a 15-ms stimulus eccentricity effect. The obtained eccentricity effect indicates that the stimulation position influenced the polarities of response codes. According to the polarity correspondence principle (Proctor and Cho, 2006), the response that corresponds to the relative position of the response set is positively coded. Even though this experiment manipulated not the response position but the stimulation position, the additional spatial code provided by the stimulation position contributed to the overall polarity of the response alternatives, resulting in opposite patterns of the polarity correspondence. That is, it is evident that polarity correspondence is modulated not only by response eccentricity but also by stimulus eccentricity. However, it should be noted that the magnitude of the stimulus eccentricity effect (15 ms) was smaller than that of the response eccentricity effect (49 ms) of Proctor and Cho’s (2003) Experiment 1. The reduction in the size of the effect was expected considering that the polarities of the response codes were modulated by stimulation position in Experiment 3 but response position in orthogonal SRC tasks. In other words, the response polarities were more strongly influenced by a spatial feature of responses (response position) than that of stimuli (stimulation position).

## Experiment 4: Timbre-Judgment Task with Eccentricity Manipulated

In Experiment 3, the correspondence effect between loudness and response side was found as a function of stimulation position when loudness was task-relevant. The correspondence effect was larger when the sound was given to the right ear (9 ms) than to the left ear (–6 ms); A 15-ms eccentricity effect was obtained. The aim of Experiment 4 was to investigate whether the eccentricity effect is present even when loudness is irrelevant to the task. In Experiment 4, a timbre-judgment task was performed with the stimulation position manipulated. In other words, participants responded to the timbre of sound, whether it was a piano tone or a violin tone, while both loudness and the ear to which the sound was given were manipulated. Even though it has been suggested that the effect of orthogonal SRC is reduced when the stimulus properties are irrelevant to the task (Wallace, 1971, 1972; Proctor et al., 2003), a trend of an eccentricity effect was expected if polarity codes are formed for the stimulation position.

### Materials and Methods

#### Participants

Sixteen new undergraduate students (mean age = 22.5, 10 females) at Korea University participated for payment of KRW 5,000 (about 4 US dollars). Four had no prior musical experience; the other twelve had an average of 6.66 years of musical training and had stopped it since 12.16 years of age on average. All were right-handed and had normal hearing as determined by self-report.

#### Apparatus, Stimuli, and Procedure

The apparatus, stimuli, and procedure were identical to Experiment 2, except as noted. The stimuli consisted of a low-level loudness tone (58-dB, SPL) and a high-level loudness tone (77-dB, SPL). Each tone was synthesized with piano and violin timbre for a total of four different stimuli. The target tones were given to the participants unilaterally through PC convertible headphones. Each tone was presented only to the left or right ear randomly. The referent tone was an intermediate-level loudness pure tone (66-dB, SPL) which was presented bilaterally. Before the experiment began, participants heard the stimuli and determined that they can distinguish the piano and violin tones. The experiment consisted of 32 practice trials and 640 test trials total. Participants performed the timbre-judgment task and timbre-to-response mapping was counterbalanced across participants. Participants were told to ignore the location of the sound presented.

### Results

0.70% of trials were removed from analyses using the same RT cutoff criteria as in the previous experiments. Mean correct RT and PE were calculated for each participant as a function of loudness, response side, and stimulation position. ANOVAs were conducted on the mean RT and PE data, with loudness (low- or high-level loudness), response side (left or right), and stimulation position (left or right) as within-subject variables, and timbre-to-response mapping as a between-subject variable. Mean RT and PE data are shown in **Table 4**.

**Table 4:** Mean RT (in milliseconds), PE, and stimulus and response polarities in parenthesis in Experiment 4 as a function of loudness, response side, and stimulation position.

	Left ear				Right ear			
Response side	Low-level loudness		High-level loudness		Low-level loudness		High-level loudness	
	RT (SD)	PE (SD)	RT (SD)	PE (SD)	RT (SD)	PE (SD)	RT (SD)	PE (SD)
Left	411 (74.26)	1.73 (2.34)	399 (71.23)	1.45 (2.95)	435 (74.39)	4.93 (6.25)	437 (73.59)	6.68 (9.39)
	(-,+)		(+,+)		(-,-)		(+,-)	
Right	435 (74.39)	5.73 (4.26)	422 (72.39)	4.43 (3.66)	397 (67.04)	2.13 (2.01)	385 (65.01)	0.88 (1.12)
	(-,-)		(+,-)		(-, +)		(+,+)	
Correspondence effect	24	4.00	-23	-2.98	-38	-2.80	52	5.81

#### RT Analysis

**FIGURE 6:**
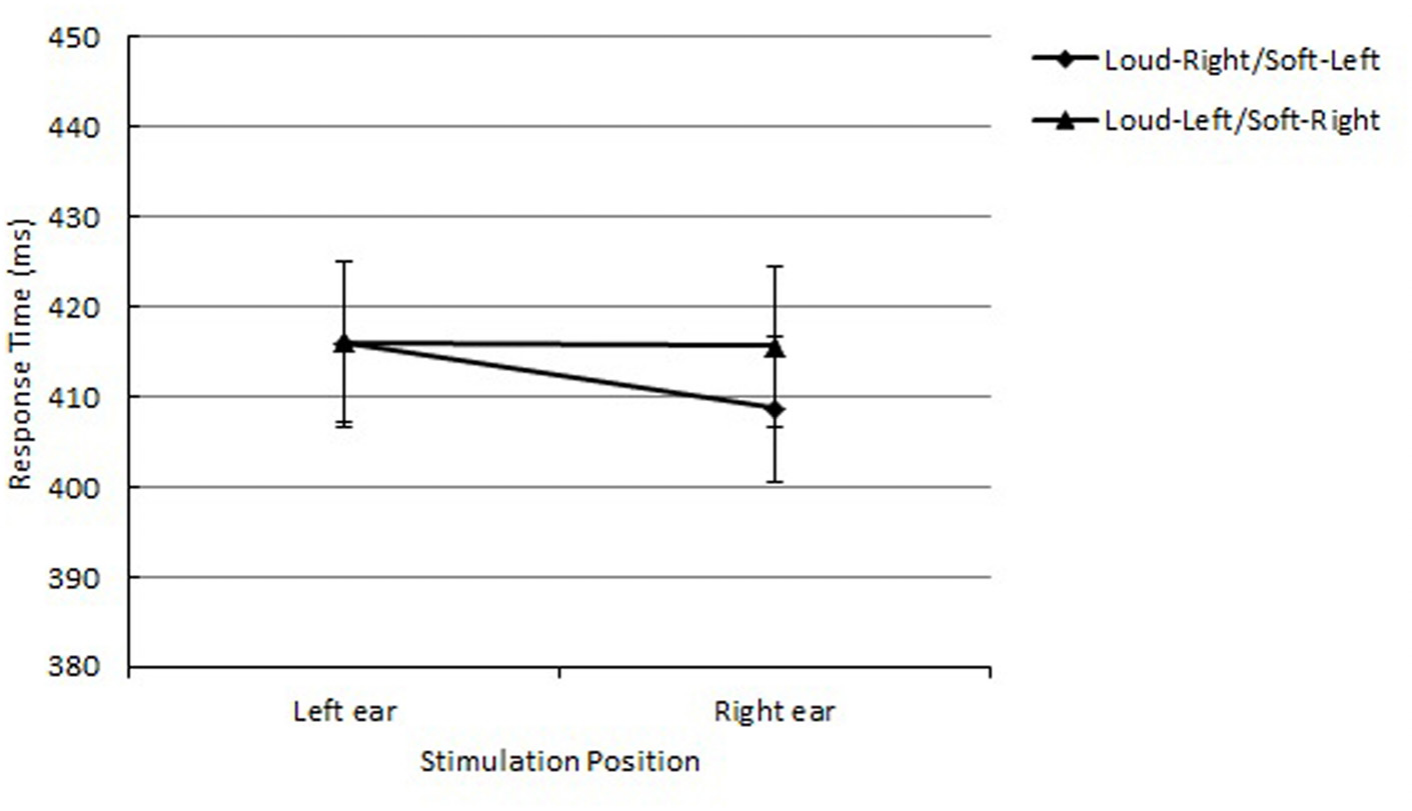
**Mean RTs as a function of loudness-response mapping and stimulation position in Experiment 4 are shown along with their SE**.

Analyses of variance revealed the main effect of loudness, *F*(1,14) = 12.36, *p* = 0.0034, *MSE* = 204, $\eta_{p}^{2}$ = 0.56. Faster RTs were obtained with high-level loudness tones (*M* = 411 ms) than low-level loudness tones (*M* = 420 ms). The interaction between stimulation position and response side was significant, *F*(1,14) = 71.00, *p* < 0.0001, *MSE* = 535, $\eta_{p}^{2}$ = 0.95, reflecting the auditory Simon effect. When the stimulation position and response side were matched, the mean RTs were shorter (*M* = 398 ms) than the non-matching mapping (*M* = 432 ms). The interaction between loudness and stimulation position was also significant, *F*(1,14) = 7.86, *p* = 0.0141, *MSE* = 58, $\eta_{p}^{2}$ = 0.19. When the sound was presented to the right ear, high-level loudness tones (*M* = 411 ms) elicited faster responses than low-level loudness tones (*M* = 416 ms). When the sound was presented to the left ear, high-level loudness tones (*M* = 411 ms) elicited faster responses than low-level loudness tones (*M* = 423 ms). Even though the 3-way interaction of loudness, response side and stimulation position was only marginally significant, *F*(1,15) = 3.11, *p* = 0.0996, *MSE* = 138, $\eta_{p}^{2}$ = 0.18, the tendency was similar to the results from Experiment 3; when the sound was presented to the right ear, the correspondence effect was greater (8 ms) than when the sound was given to the left ear (0 ms; see **Figure 6**). Any effects regarding timbre-to-response mapping did not reach significance, indicating that it is not a critical factor for the correspondence effect between loudness and response side.

#### PE Analysis

The overall PE was 3.49%. Only the interaction between response side and stimulation position was obtained, *F*(1,14) = 17.60, *p* = 0.0009, *MSE* = 28, $\eta_{p}^{2}$ = 0.88, indicating an auditory Simon effect. When the sound location and response side matched (1.55%), the performance was better than when they did not (5.44%). All the other effects did not reach significance.

### Discussion

When participants were required to respond to the timbre rather than the loudness, a marginally significant eccentricity effect was obtained. The correspondence effect was larger when the sound was presented to the right ear (8 ms) than to the left ear (0 ms). The obtained stimulus eccentricity tendency is in line with the polarity correspondence principle. Even though the loudness of sound was irrelevant to the task, stimulation position as well as loudness of tones and response sides was categorically coded as + or - polarity, respectively. As discussed in Experiment 3, the eccentricity of stimulation position influenced the polarities of the responses, which elicited the stimulus eccentricity effect.

However, the stimulus eccentricity effect was at most half the size of the effect obtained in Experiment 3 in which loudness was relevant to the task. This difference in the effect sizes is in agreement with results found in the previous orthogonal SRC experiments in which the size of orthogonal SRC effects was smaller when stimulus location was irrelevant to the task than when it was task-relevant (Wallace, 1971, 1972; Proctor et al., 2003) and the magnitude of response eccentricity effects also showed a similar tendency (Cho and Proctor, 2002, 2004; Nishimura and Yokosawa, 2006; Cho et al., 2008). The previous studies reported that the size of the response eccentricity effect was smaller when stimulus location was task-irrelevant (25–28 ms; Nishimura and Yokosawa, 2006; Cho et al., 2008) than when it was relevant to the task (60–80 ms; Cho and Proctor, 2002, 2004). Even though the stimulus and response alternatives are coded as + or - polarity and the correspondence of the polarities influences response selection even when stimulus location or loudness is task-irrelevant, the effect is not as clearly evident as when stimulus location or loudness is relevant to the task. The finding that no effect regarding timbre-to-response mapping was found demonstrates timbre is not a critical factor for the obtained correspondence effect and stimulus eccentricity effect.

## General Discussion

### Primary Outcomes and Polarity Coding Underlying Auditory Orthogonal SRC Effects

Consistent with the predictions based on the polarity correspondence principle, a significant correspondence effect was found between loudness and response side regardless of task relevance (Experiments 1 and 2). According to the principle, the polarity correspondence effect occurs because stimulus and response alternatives are coded asymmetrically with one of two alternatives for each dimension being categorically coded as + polarity and the other as -. That is, the correspondence of polarity codes elicited by stimulus and response alternatives are core determinants for the orthogonal SRC effects (Proctor and Cho, 2006) and the SMARC effect, which is a variant of the orthogonal SRC effect (Rusconi et al., 2006; Nishimura and Yokosawa, 2009; Cho et al., 2012). It has been suggested that “up” is coded as + and “down” as - when the alternatives vary along the vertical dimension, while “right” is coded as + and “left” as - when they vary along the horizontal dimension. This is because “up” and “right” serve as the polar referent in each dimension and “down” and “left” are coded in relative to their respective referents as a result of asymmetric coding (Clark and Chase, 1972; Olson and Laxar, 1973, 1974; Seymour, 1974a,b; Carpenter and Just, 1975). Therefore, according to the polarity correspondence principle, the correspondence effect between loudness and response side is obtained because the polarity codes for “loud” and “right” (+) and the codes for “soft” and “left” (-) correspond, respectively.

Furthermore, the finding of the correspondence effect even when the loudness was irrelevant to the task in Experiment 2 implies the possibility of the polarity codes for loudness influencing the response selection processes in the previous experiments that show inconsistent results on the horizontal SMARC effect, in which the pitch height was irrelevant to the task. It is possible that trial to trial variations in loudness influence pitch judgments and that variation in frequency influences judgments on loudness. This is a type of *Garner interference* (Garner and Felfoldy, 1970). To elaborate, orthogonal variation on the irrelevant dimension causes interference with the classification of the relevant dimension when two dimensions interact. Numerous studies that have used a tone, a multidimensional stimulus that consists of pitch loudness and timbre, have shown that pitch and loudness dimensions interact in an integral fashion (e.g., Grau and Kemler-Nelson, 1988). Being relevant to this issue, it should be noted that previous studies on the SMARC effect did not control for or take *equal loudness contours* into consideration when pitch was manipulated. According to the equal loudness contours for the human ear, when the sounds are at different frequencies the sensitivity of our hearing varies as the frequency varies (Fletcher and Munson, 1933). In other words, sounds with equal intensities but different frequencies are perceived to differ in loudness. For example, a 1,000 Hz sound at 60 dB is perceived louder than a 500 Hz sound at 60 dB. Furthermore, even though the perceived loudness is related to its amplitude, loudness, and amplitude do not have a simple linear–functional relationship. Therefore, it has been recommended to use an instrument called a sound level meter, to measure loudness and compensate for the variation of sensitivity of the ear as a function of frequency by weighting frequency (Howard and Angus, 2009). Considering the equal loudness contours and the suggestions for loudness measurement, the unequalized or inadequately equalized loudness during the variation of frequency might have made the perceived loudness of stimuli fluctuate and the variation in loudness might have influenced pitch judgments in the previous experiments as a type of Garner interference. That is, with regard to the interactive relation between pitch and loudness, it is highly probable that the variations in loudness, which was an irrelevant attribute, interfered with pitch judgments in previous studies. Hence, concerning that the loudness is also categorically coded relative to a reference point on the loudness dimension resulting in polarity coding, both pitch and loudness could have elicited polarity correspondence effects with lateralized responses respectively in the previous studies. It is plausible that different levels of loudness led the high or low pitch tones to be coded as loud (+) or soft (-), thus attenuating the SMARC effect.

In contrast, the spatial mental representation account emphasizes a spatial correspondence between tones being located on the vertical or horizontal pitch line and the up/right or down/left response dimension, respectively. Regarding the horizontal SMARC effect, Lidji et al. (2007) suggested that musicians’ knowledge of using keyboard helps representing pitch height on the keyboard-like horizontal line automatically. They attributed the absence of horizontal SMARC effect in non-musicians to their inability to automatically represent the pitch height on to a mental horizontal line. In a similar way, because of a population stereotype of loudness control in which high-level loudness is associated with a rightward shift and low-level loudness with a leftward shift, the correspondence effects between loudness and response side were possibly obtained in Experiments 1 and 2. However, the findings of the correspondence effects obtained as a function of stimulation position in Experiments 3 and 4 are hardly explicable with the spatial mental representation account.

A significant stimulus eccentricity effect was found when loudness was task-relevant in Experiment 3 and a marginally significant stimulus eccentricity effect was obtained when loudness was irrelevant to the task in Experiment 4. The eccentricity effect has been mainly studied with the manipulation of the response-set position along the horizontal dimension in the orthogonal SRC task, resulting in the response eccentricity effect (e.g., Michaels, 1989). In the current study, the first attempt was made to reveal an eccentricity effect by manipulating stimulation position along the horizontal dimension. It was assumed that the stimulation position (ear) to which auditory sound was presented was spatially coded relative to the body midline. For example, when sound is presented to the right ear, the formed spatial code “right” provides an additional + code, resulting in an evident loud-right/soft-left advantage. On the other hand, when sound is presented to the left ear, “left” is coded as + polarity because of the additional “left” spatial code from the stimulation position, resulting in a less evident loud-right/soft-left advantage or a loud-left/soft-right advantage. In other words, the response code corresponding to the stimulated ear earns + polarity regardless of whether it is “right” or “left” whereas the response code corresponding to the unstimulated ear earns - polarity, simultaneously changing the polarity codes of responses depending on the newly obtained spatial code. As a result, an eccentricity effect emerges as a function of the stimulation position. However, it should be noted that the Simon effect was evident between the stimulated ear and response side in Experiments 3 and 4 regardless of polarity codes. Thus, some might argue that the eccentricity effects resulted possibly from the Simon congruency. To test this possibility, additional statistical analyses were conducted with only Simon corresponding trials in Experiments 3 and 4. The results showed that RT was significantly faster on polarity corresponding trials than polarity non-corresopnding trials both in Experiment 3, *F*(1,15) = 8.88, *p* = 0.0093, and Experiment 4, *F*(1,15) = 14.67, *p* = 0.0016, indicating that the obtained stimulus-eccentricity effects were due to polarity correspondence.

Overall, as predicted, the results showed that the correspondence effect was larger when the sound was presented to the right ear than when it was presented to the left ear in both Experiments 3 and 4. This pattern of results indicates that the polarity codes of response alternatives can be influenced by a feature of stimulus (e.g., stimulation position), as well as features of response (e.g., response position). For visual tasks, the polarity correspondence effect varied as a function of the response position relative to visual stimuli sets (Cho and Proctor, 2005), whereas for the auditory tasks in the present study, the effect varied as a function of the stimulation position. This disparity is due to the difference between the natures of the visual and auditory perceptual processes, as demonstrated by the findings that separate neural subsystems are involved in auditory and visual spatial localizations (e.g., Bushara et al., 1999) and that conceptual representations as well as perceptual representations have their bases on modality specific systems (e.g., Barsalou et al., 2003). That is, when a visual stimulus is presented, the response position relative to it is evident. However, when an auditory stimulus is presented, it is difficult to use the stimulus as a referent point for the response position, even though the spatial code for the stimulation is clearly formed.

It is important to note that Santiago and Lakens (2015) failed to obtain the response eccentricity effect in a parity judgment, a magnitude judgment, and a time categorization task when the keyboard position was manipulated. The authors indicated that conceptual congruency effects of both number and time dimensions with the left-right spatial dimension were not probably due to the polarity correspondence. However, Lakens (2012) discovered the modulation of the polarity correspondence in a conceptual categorizing task by adopting a training task in which the relative frequencies of + and - polarity words were manipulated. As Lakens (2012) and Santiago and Lakens (2015) suggested, one possible reason for these inconsistent findings of the modulation of the polarity correspondence effect is that a strong manipulation is necessary to change the polarity structure of the conceptual dimensions.

### The Possibility of Polarity Correspondence Principle as An Integrated Framework

A variety of non-spatial SRC effects including the aforementioned SMARC effect have been found and the underlying mechanisms have been widely investigated. For example, when people perform a parity judgment task, the asymmetries between numbers and responses exert two effects: the *linguistic markedness association of response codes* (*MARC) effect* and the *SNARC effect*. The MARC effect refers to the phenomenon in which performance is better for the even-right/odd-left mapping than for the opposite mapping (Hines, 1990; Reynvoet and Brysbaert, 1999; Cho and Proctor, 2007). The MARC effect has been attributed to the linguistic markedness because the correspondence of the unmarked (even) and marked (odd) verbal codes with the unmarked (right) and marked (left) response codes yields better performance (Hines, 1990; Nuerk et al., 2004; Cho and Proctor, 2007). The SNARC effect refers to better performance yielded when a large number is associated with the right response and a small number with the left response than the opposite association. Many researchers believe that these non-spatial SRC effects are due to the spatial aspect of the mental representations of sequence information, as the spatial mental representation account suggested. According to this account, there is a horizontal line on which non-spatial sequence information is represented from left to right along the horizontal axis in space, and the spatial correspondence between stimulus and response dimension elicits the SRC effects (Dehaene et al., 1993; Lidji et al., 2007).

However, those non-spatial SRC effects have been also shown to conform to the polarity correspondence principle. As mentioned earlier, the SMARC effect is obtained because the polarity of a high pitch tone (+) corresponds with up or right (+) response and that of a low pitch tone (-) with down or left (-) response. Furthermore, the polarity correspondence principle suggests that the parity and the magnitude of numbers are coded asymmetrically with even or large numbers being coded as + polarity and odd or small as - polarity. This would lead to the MARC or SNARC effect when the right (+) and left (-) responses are associated with even/large (+) and odd/small (-) numbers. The findings that Arabic numerals as well as digit words yielded the MARC effect, even though the size of the effect was larger for digit words than Arabic numerals (Nuerk et al., 2004), indicate that the MARC effect is not restricted to verbal codes. Furthermore, the findings that the SNARC effect was larger after practice with up-right/down-left mapping than up-left/down-right mapping but being not affected by whether participants practiced with a parallel SRC task with compatible or incompatible mapping (Bae et al., 2009), provide a possibility that the SNARC effect is due to polarity coding.

Even though various non-spatial SRC effects such as the SMARC, the MARC, and the SNARC effects have been found in diverse domains and explained in their own ways, the polarity correspondence provides an integrated framework encompassing those various non-spatial SRC effects as well as the correspondence effect between loudness and response side, which is the major finding of the current research. The evidences from previous studies and the current study demonstrate that the polarity correspondence principle plays a fundamental role in process of response selection in binary choice tasks in general. Therefore, when a variety of issues in psychological research that involves the use of binary choice tasks is investigated, researchers should be aware of how polarity coding and correspondence operates and should rule out the possibility of polarity coding of irrelevant dimension influencing the obtained results.

## Conflict of Interest Statement

The authors declare that the research was conducted in the absence of any commercial or financial relationships that could be construed as a potential conflict of interest.
